# Hygroscopic Porous Polymer for Sorption‐Based Atmospheric Water Harvesting

**DOI:** 10.1002/advs.202204724

**Published:** 2022-10-09

**Authors:** Fangfang Deng, Zhihui Chen, Chenxi Wang, Chengjie Xiang, Primož Poredoš, Ruzhu Wang

**Affiliations:** ^1^ Institute of Refrigeration and Cryogenics MOE Engineering Research Center of Solar Power and Refrigeration Shanghai Jiao Tong University Shanghai 200040 China

**Keywords:** hygroscopic porous polymer, sorption‐based atmospheric water harvesting, sorption kinetics, sorption mechanism, structural optimization

## Abstract

Sorption‐based atmospheric water harvesting (SAWH) holds huge potential due to its freshwater capabilities for alleviating water scarcity stress. The two essential parts, sorbent material and system structure, dominate the water sorption–desorption performance and the total water productivity for SAWH system together. Attributed to the superiorities in aspects of sorption–desorption performance, scalability, and compatibility in practical SAWH devices, hygroscopic porous polymers (HPPs) as next‐generation sorbents are recently going through a vast surge. However, as HPPs’ sorption mechanism, performance, and applied potential lack comprehensive and accurate guidelines, SAWH's subsequent development is restricted. To address the aforementioned problems, this review introduces HPPs’ recent development related to mechanism, performance, and application. Furthermore, corresponding optimized strategies for both HPP‐based sorbent bed and coupling structural design are proposed. Finally, original research routes are directed to develop next‐generation HPP‐based SAWH systems. The presented guidelines and insights can influence and inspire the future development of SAWH technology, further achieving SAWH's practical applications.

## Graphical abstract

Hygroscopic porous polymers (HPPs) as novel sorbents have excellent water sorption performance, wide‐humidity applicability, and high system compatibility. Effective structural optimizations of sorbent beds and other components enable HPP‐based SAWH systems to exhibit desirable dynamic performance even with large material dosage. It can inspire the design of next‐generation SAWH system with promising water yield, further addressing the water shortage problem.

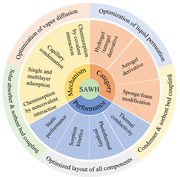

## 1 Introduction

With the occurrences of climate change and water supply pollution, drought has become a severe threat to people's survival.^[^ ^1^ ^]^ Up to 2050, freshwater will be inaccessible for more than 2 billion people owing to the contradiction between limited freshwater resources and the surge of water requirements.^[^ ^2^, ^3^ ^]^ To alleviate the water scarcity stress, some technologies such as seawater desalination and atmospheric water harvesting (AWH) are urgently explored.^[^ ^4^, ^5^ ^]^ Among them, seawater desalination relying on seawater is inapplicable in landlocked regions where a relatively higher share of the population lacks freshwater. In this case, AWH technology based on the atmospheric air which accounts for 10% of all other freshwater resources is a more universal and diverse source of water in addressing the global water problem.^[^ ^6^ ^]^ Corresponding methods mainly include fog capture, dew collection, condensation based on refrigeration, and sorption‐based atmospheric water harvesting.^[^ ^7^ ^]^ Although fog capture and dew collection have been used for military and post‐disaster freshwater supplies, it is impractical when relative humidity is below 40%. The condensation process based on refrigeration, on the other hand, is highly energy‐intensive in low or medium humidity regions.^[^ ^8^ ^]^ In comparison, SAWH is the only technology that can provide clean water for all humidity ranges even in extremely water‐scarce areas in an energy‐efficient manner. Thus, considering the wide applicability and high efficiency, SAWH is distinguished from all kinds of AWH technologies.^[^ ^9^, ^10^ ^]^

**Figure 1:**
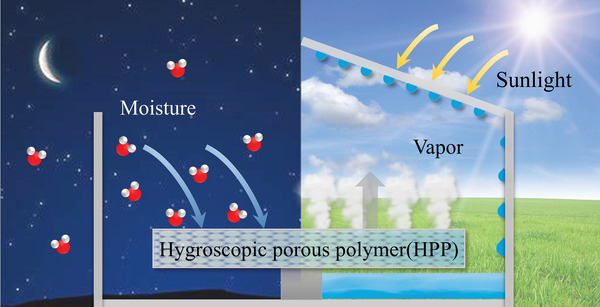
Components and operational mechanism for HPP‐based SAWH system.

Considering the SAWH technology, sorbent is one of the crucial elements for exerting functions of moisture sorption and desorption (**Figure** **1**). Conforming to the development of SAWH technology, various sorbents have emerged subsequently, such as conventional sorbents (silica gel, zeolite), composite sorbents (CaCl_2_‐impregnated silica gel), metal–organic frameworks (MOFs), hydrogels, and other original composites.^[^ ^11^, ^12^ ^]^ Depending on their individual properties, various sorbents present different sorption performances and suit the specific operational condition. Certain sorbents are desirable candidates for sorption‐based water harvesting under different conditions. In desert regions, MOFs such as MOF‐801, MOF‐303, and some hygroscopic polymers such as calcium alginates are usually selected to extract freshwater.^[^ ^6^, ^13^, ^14^, ^15^ ^]^ In semi‐arid and semi‐humid regions, composites such as carbon nanosphere impregnated with LiCl, composed hydrogels such as PAM‐CNT‐CaCl_2_, and hygroscopic polymers such as sponge‐based CaCl_2_‐included complex were found to have a satisfactory performance.^[^ ^16^, ^17^, ^18^ ^]^ In humid regions, some pure hydrogels can realize considerable water uptake.^[^ ^19^ ^]^

Nevertheless, due to the weak hygroscopicity at low humidity, many pure hydrophilic polymers are overshadowed for water harvesting in low‐humidity regions. The deliquescent phenomenon at high humidity prevents some salts‐impregnated composites from exhibiting desirable sorption performance in practical SAWH with unsteady humidity conditions. Restricted by the powdery form of MOFs, those materials are a disputable choice in practical SAWH devices, which requires to be stacked to impair sorption kinetic.^[^ ^20^ ^]^ The worse heat and mass transfer properties of MOF‐based sorbent bed will impair integral water harvesting performance and degrade water production of a practical SAWH device. Owing to the above problems from sorbent to SAWH system, a sharp attenuation of water harvesting performance often occurs when utilizing these sorbents in real SAWH devices. Taking it into consideration, some hygroscopic porous polymers (HPPs) such as gel, gel's derivative, and foam/sponge's modification have been developed and reported.^[^ ^21^, ^22^, ^23^, ^24^ ^]^ Benefiting from their versatile sorption mechanisms and unique structural characteristics, HPPs possess high water capacity, fast sorption kinetics, and high thermal conductivity compared to other novel sorbents. In addition, the form of scalable monolith makes HPPs exceptional at exerting desirable SAWH performance in a practical device without sacrificing the material's performance. Compared with powdery MOFs or granular composites, HPPs can overcome the bottleneck to ensure a compelling performance from material to system levels, which marks a potential to promote SAWH's marketization.^[^ ^25^ ^]^

Recently, many promising HPPs are reported for SAWH applications. According to their different sorption mechanisms, HPPs can be mainly classified into two categories: one is endowed with SAWH capacity relying on surficial hydrophilic functional groups and porous structure with coarse surface; the other is the combination of a porous skeleton (porous skeletons include hydrogel, aerogel, foam, sponge, etc.) and hygroscopic factor (hygroscopic factors include some inorganic hygroscopic salts, organic hydrophilic polymers, and other active ions, both of which can capture moisture strongly, relying on their high affinity to water vapor, for e.g., CaCl_2_, LiCl, CoCl_2_, [EMIM][AC], PPy‐Cl, glycerin, etc.). Typical HPPs belonging to the first mechanism are some pure hydrogels or aerogels, such as ZnO*_x_* hydrogel and G‐PDDA aerogel.^[^ ^26^, ^27^ ^]^ The second kind of HPPs embeds inorganic salts or organic hydrophilic monomers in gel‐based or foam/sponge‐based skeletons, such as PAM‐CNT‐CaCl_2_ and LiCl@rGO‐SA.^[^ ^18^, ^21^ ^]^ However, their detailed sorption mechanism, performance, and applied potential lack comprehensive and accurate guidelines. Concerning subsequent SAWH progress as well as the development of next‐generation HPP, a thorough understanding related to the HPP‐based SAWH system is of utmost necessity, which is the main topic of this review. In Section 1, we introduce the background of SAWH technology, sorbent's development, and HPPs’ current development in brief. Section 2 illustrates HPPs’ sorption mechanisms in detail, while Section 3 deals with the working principle of HPP‐based SAWH as well as HPPs’ static and kinetic sorption–desorption performance. Section 4 proposes the optimized strategies aiming at the HPP‐based SAWH system, mainly in terms of the HPP‐based sorbent bed and the coupling design for all systemic components. Section 5 promotes the outlook on how to design next‐generation HPP, HPP‐based sorbent bed, and relevant hybrid application.

## 2 Water Sorption Mechanism and Behavior

**Figure 2:**
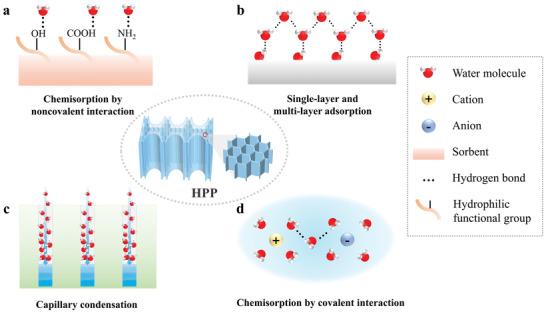
Sorption mechanism for HPPs.

HPPs’ water sorption often occurs following four kinds of mechanisms: i) chemisorption by noncovalent interaction between hydrophilic functional groups and water molecules; ii) single‐layer and multi‐layer adsorption in the form of layers or clusters; iii) capillary condensation in the nanoscale porous structure; and iv) chemisorption by covalent interaction between hygroscopic factors and water molecules (**Figure** **2**). According to the above mechanisms, gel‐based and foam‐based HPPs exhibit two kinds of water sorption behavior: one mainly relies on the physisorption process based on mechanisms (i–iii); the other mainly depends on the hygroscopic factor with the mechanisms (i,iv).

### 2.1 Chemisorption by Noncovalent Interaction

HPPs are typically based on porous substrates such as hydrogel, aerogel, foam, and sponge, most of which are synthesized by crosslinking, copolymerization, freeze‐drying, and foaming methods.^[^ ^28^, ^29^, ^30^ ^]^ Some organic monomers or polymers with numerous functional groups are generally utilized to participate in the polymerization process. Through the cross‐linking process, a stable network of the polymeric sorbent is built, of which there are some functional groups such as hydroxyl (—OH), amino (—NH_2_), carboxylic acid (—COOH), and sulfonic acid (—SO_3_H) groups.^[^ ^31^ ^]^ These hydrophilic functional groups possess lone‐pair electrons and vacancies, which could be regarded as the donor and acceptor for surrounding water molecule to establish hydrogen bonds.^[^ ^32^ ^]^ When exposed to atmospheric moisture, these hydrophilic functional groups bond with water vapor molecules through hydrogen bonds or electrostatic interaction,^[^ ^33^ ^]^ enabling HPP to exhibit water sorption behavior. The layer of captured water associates with HPPs’ polymer chain via noncovalent interaction and belongs to the bound water. Formed by HPPs’ hydroxylation, the bound water appears at a slightly higher binding energy. Changing the proportion of raw monomers or polymers^[^ ^34^ ^]^ can tune the amount of hydrophilic functional groups on HPP, further affecting the amount of non‐freezable bound water and HPPs’ water sorption capacity.

### 2.2 Single‐Layer and Multi‐Layer Adsorption

According to the absorption behavior, as discussed in Section 2.1, an ordered configuration of one‐layer absorbed water molecules orients to form on HPPs’ surface via the noncovalent interaction of hydrophilic functional groups. It induces the distinct varied dipole moment for this part of water molecules, which further acts as additional adsorption active sites.^[^ ^35^ ^]^ With the rise of water vapor partial pressure on HPPs’ surface, neighboring water vapor molecules bond with the previous layer of absorbed water.^[^ ^36^ ^]^ The physisorption behavior is the multi‐layer adsorption, which relies on the van der Waals forces.^[^ ^37^ ^]^ As the water captured by multi‐layer adsorption exists between the water captured by HPPs’ hydrophilic functional groups and the free water captured in subsequent sorption, it is generally named intermediate water. The intermediate water shows relatively weaker binding energy compared with the foregoing bound water.^[^ ^38^ ^]^ HPPs’ crosslinking density, network structure, and the amount of hydrophilic functional group not only have a significant effect on its water sorption capacity but also determine the proportion of intermediate water, bound water, and free water.^[^ ^39^ ^]^ Modifying HPP to promote the percentage of intermediate water can reduce HPPs’ integral desorption enthalpy, which is beneficial for the accomplishment of HPPs’ desorption process driven by low‐grade energy.^[^ ^40^ ^]^

### 2.3 Capillary Condensation

Capillary condensation is governed by the Kevin equation, describing that the water vapor can be condensed in a capillary channel even though the water vapor partial pressure is lower than the saturated value, if reaching the critical pore diameter.^[^ ^41^ ^]^ As for the mesopores (with a diameter of 2–50 nm)^[^ ^42^ ^]^ in HPP, the surface tension on concave adsorbed water film can exert an additional generalized force to complement the required molar power for water vapor's condensation process.^[^ ^43^ ^]^ When the pore diameter nearly equals the critical Kevin pore diameter, the capillary condensation phenomenon occurs. This kind of sorption mechanism enables HPP to achieve considerable water uptake, especially at high relative humidity.^[^ ^27^ ^]^ Attributed to freeze‐drying, electrospinning, and foaming methods, HPPs can be synthesized with a nanoscale porous structure with an enormous surface area.^[^ ^39^ ^]^ Experiencing the absorption and multi‐layer adsorption mentioned in Section 2.3, some macropores (with a diameter of more than 50 nm) can also contribute to the capillary condensation and harvest the water vapor.^[^ ^20^ ^]^ Captured water via capillary condensation generally belongs to the free water, which mainly interacts with adjacent water molecules rather than HPPs’ network. The number of nanoscale pores and the cross‐link density of HPP decide its water uptake derived from capillary condensation, as well as affect the relative share of free water.^[^ ^27^ ^]^ Due to the different water vapor partial pressures in pores during sorption and desorption, there is a hysteresis loop on HPPs’ water sorption–desorption isotherm.

### 2.4 Chemisorption by Covalent Interaction

Different from noncovalent interactions such as hydrogen bonds and electronic interactions, covalent interactions mainly include ionic bonds and coordination bonds. Referring to HPPs with the chemisorption behavior, some hygroscopic factors impregnated in HPPs’ network structure could capture and liquefy atmospheric water vapor via ionic interaction or coordination effect. Unlike physisorption that happens on the sorbent's surface, this chemisorption usually induces water incorporated into the volume of HPP.^[^ ^24^ ^]^ Taking hygroscopic inorganic salts (CaCl_2_, LiCl, ZnCl_2_, etc.) as an example, first, the salts capture bits of water vapor and form coordination bonds between salt crystal's vacancies and absorbed water molecules.^[^ ^44^, ^45^ ^]^ Further, the continuous water harvesting process induces the solvation process of salt crystals, which indicates that subsequent absorbed water interacts with salts’, both anions and cations by ionic bonds, hydrogen bonds, and electrostatic interactions. With the formation of a stable hydration layer, consecutive water vapor is captured by the concentrated salty solution until the water vapor's partial pressure on the salty solution's surface is equal to on the ambient's. Last, in the HPP, salty solution is composed of countless spherical clusters which are water molecules surrounding anions or cations. The salty solution is encapsulated in HPPs’ porous network structure, while it permeates inside depending on the osmosis pressure. The absorbed water is dissociative and hardly associates with HPPs’ polymer chain and belongs to the free water. It must be mentioned that sufficient salty solution in porous structure could prevent the deliquesce and leakage attributed to HPPs’ capillary force, especially for hydrogel‐based HPP with excellent swelling properties. Correspondingly, the content of hygroscopic factors could primarily determine HPPs’ water sorption capacity. In addition, the chemisorption via covalent interaction makes a higher energy barrier compared with other sorption mechanisms, owing to the high binding energy for ionic or coordination interaction (≈450 kJ mol^−1^).^[^ ^46^ ^]^

### 2.5 Two Kinds of Water Sorption Behaviors

Most HPPs exhibit comprehensive water sorption behavior rather than a single sorption mechanism. According to the above four kinds of mechanisms, HPPs could be mainly classified into two categories of sorption behaviors. The first one mainly includes some pure hydrogels and aerogels, which utilize surficial hydrophilic functional groups to capture the first layer of well‐ordered water molecules.^[^ ^47^ ^]^ The part of bound water as the additional active sites induces the multi‐layer adsorption behavior, followed by the capillary condensation in numerous nanoscale pores.^[^ ^48^ ^]^ Such HPPs often appear wrinkled with rough nanoscale porous structures. They furnish countless hydrophilic sites to capture vast water molecules, enormous surface area to guarantee sufficient multilayer adsorption, and valid nanoscale pores to conduct considerable capillary condensation.^[^ ^26^, ^27^ ^]^ The second one is mainly the combination of the hygroscopic factor and the light porous skeleton. For this kind of HPP, a hygroscopic factor can proceed with the chemisorption process and capture moisture by forming hydrogen bonds, electronic interactions, or covalent interactions.^[^ ^49^, ^50^ ^]^ The porous substrate is responsible for storing and encapsulating the harvested water.^[^ ^51^ ^]^ The unique combination of the two components enables HPP to realize extraordinary water sorption performance in full humidity ranges.

## 3 Sorption and Desorption Performance

### 3.1 Working Principle of HPP‐Based SAWH System

A desirable SAWH system integrating HPP, condenser, and other components is generally driven by a low‐grade, easily available, and sufficient energy source, such as solar energy. HPP as the core component of the SAWH system is dominantly responsible for the sorption and desorption processes. With the help of a condenser and water collector, a subsequent condensation process could occur to produce freshwater. As shown in Figure 1, the main water harvesting process consists of three steps: i) HPP captures water vapor from the atmosphere under operational conditions (below 40 °C); ii) HPP desorbs hot water vapor to ambient air at elevated temperature and low relative humidity; and iii) the desorbed hot moisture releases heat and condenses into water droplets. As a promising sorbent for SAWH technology, HPP is required to possess the following qualities: i) considerable water uptake capacity under operational conditions; ii) fast sorption kinetics in a time‐limited single cycle; iii) low energy requirement and appropriate desorption temperature (below 100 °C); iv) physical and chemical stabilities for long‐term operation; and v) cost effectiveness and synthesis scalability. Among them, HPPs’ static and kinetic performances will be highly discussed due to their pivotal influences on systemic water productivity.

### 3.2 Sorption Performance of HPPs

#### 3.2.1 Static Performance

**Figure 3:**
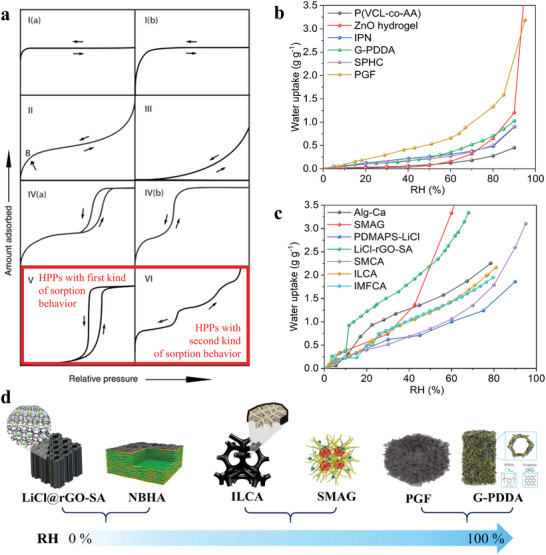
Static performance of HPPs. a) Different kinds of sorption isotherm according to the IUPAC standards. Reproduced with permission.^[^ ^52^ ^]^ Copyright 2015, De Gruyter. b) Water sorption isotherm for HPPs that follows the first kind of sorption behavior.^[^ ^26^, ^27^, ^53^, ^54^, ^55^, ^56^ ^]^ c) Water sorption isotherm for HPPs that follows the second kind of sorption behavior.^[^ ^21^, ^23^, ^24^, ^57^, ^58^, ^59^, ^60^ ^]^ d) Selection of HPPs under different relative humidity regions. LiCl@rGO‐SA in d) Reproduced with permission.^[^ ^21^ ^]^ Copyright 2021, Royal Society of Chemistry. NBHA in d) Reproduced with permission.^[^ ^22^ ^]^ Copyright 2021, Elsevier. ILCA in d) Reproduced with permission.^[^ ^59^ ^]^ Copyright 2021, Elsevier. SMAG in d) Reproduced with permission.^[^ ^23^ ^]^ Copyright 2019, Wiley. PGF in d) Reproduced with permission.^[^ ^56^ ^]^ Copyright 2019, Wiley. G‐PDDA in d) Reproduced with permission.^[^ ^26^ ^]^ Copyright 2020, Elsevier.

Static sorption performance represents a condition in which the sorption process of the sorbent has finished and got in equilibrium under a given temperature and relative humidity condition, which is not related to time. It denotes the saturated sorption capacity as well as the maximum water uptake. Commonly, water sorption isotherm is the utmost pivotal indicator for evaluating sorbent material's static performance, which can reflect its maximum potential for SAWH application under different operational conditions. The water sorption isotherm of a sorbent is tightly associated with the material's pore structure, surface topography, water affinity, and chemical components. The shape of the water sorption isotherm mainly depends on the material's sorption mechanism, which exactly refers to the specific interactions between sorbents and water molecules. For various sorbent materials, the water sorption isotherms can be divided into six types derived from the classification of the International Union of Pure and Applied Chemistry (IUPAC) referred to as the physical sorption mechanisms (**Figure** **3**a).^[^ ^52^ ^]^

Depending on the chemical properties, HPPs’ water sorption isotherms can be classified into two types: one that belongs to the abovementioned type V; the other is similar to the abovementioned type VI demonstrating the water uptake with an almost linear correlation with the increase of relative humidity. Figure 3b summarizes recently reported HPPs, whose water sorption isotherm is similar to type V. These HPPs present comparatively low water sorption capacity (<0.6 g g^−1^) at a relative humidity below 60%, while their saturated water uptake increases rapidly even up to 3.5 g g^−1^ when relative humidity is in the range of ≈60–100%. This kind of HPPs captures moisture on the following mechanisms: i) bonding of water molecule with surficial hydrophilic functional groups; ii) multilayer water adsorption upward the first layer of the water molecule; and iii) surficial condensation on a coarse surface or capillary condensation in nanoscale pores under high relative humidity ranges. Several pure hydrogels or aerogels with nanoscale porous structures follow exactly this sorption principle, such as ZnO*_x_* hydrogel, G‐PDDA aerogel, PGF aerogel, and so on.^[^ ^26^, ^27^, ^53^, ^54^, ^55^, ^56^, ^61^ ^]^ Figure 3c has listed several typical HPPs with outstanding static sorption performance over a wide humidity range. This kind of HPPs is fabricated by adding a hygroscopic factor with high water affinity into a light porous network skeleton. They follow the second kind of water sorption behavior as shown in Section 2.5. Besides its encapsulation for hygroscopic factors, the porous skeleton also provides excellent transfer channels for the diffusion of water vapor and the permeation of liquid water, which is exactly one of the significant advantages of HPP compared to other powdery sorbents.^[^ ^62^ ^]^ The dominant sorption capacity derives from homogeneously dispersed hygroscopic factors; however, the hydrophilic porous skeleton also demonstrates a fraction of accountability for water harvesting. Attributed to their superimposed effect, the water sorption isotherms of this kind of HPPs are parallel to type VI. As relative humidity increases in the range of 0–60%, HPPs’ static water sorption capacity increases almost linearly, which is more considerable compared with HPPs listed in Figure 3b. When relative humidity is higher than 60%, the slope of the sorption isotherm curve steepens, owing to the superior sorption performance of HPPs’ hydrophilic skeleton compared with that at low relative humidity. Typical HPPs such as PDMAPS‐LiCl, SMAG, and IMFCA are promising candidates for water harvesting in wide relative humidity conditions.^[^ ^23^, ^24^, ^59^ ^]^

In addition, HPPs’ water sorption isotherms could determine their suitable applied conditions (Figure 3d). As shown in **Table** **1**, we can select appropriate HPPs for practical applications referring to HPPs’ sorption capacities. Except for a few HPPs with exhibited sorption performance in a wide humidity range, most of the HPPs show outstanding performance especially under a certain humidity condition. For instance, at low relative humidity, both LiCl@rGO‐SA and NBHA are tailored for SAWH application contributing to their high hygroscopicity even in arid areas.^[^ ^21^, ^22^ ^]^ In humid areas, pure HPPs belonging to type V are appropriate, benefiting from their considerable water uptake and low energy requirement for desorption. It's worthwhile noting that the excellent swelling behavior, such as SMAG hydrogel, and the strong capillary force, as mentioned by ILCA, endow themselves with flexible water‐retaining properties. It could prevent HPP from the deliquesce and leakage phenomenon although the water uptake is quite large, which is a bright spot of HPP distinguished from other novel composite sorbents.

**Table 1:** Summary of different kinds of HPPs with their sorption performance

Type	HPP	Sorption water capacity [RH]	Sorption kinetics for micro‐scale sorbent	Ref.
Hydrogel or xerogel	Alg‐CaCl_2_	0.66 g g^−1^ (RH26%)	30 °C and RH30%: 0.8 g g^−1^ (10 h)	[57]
	PNIPAAm/Alg IPN gel	0.18‐0.31‐0.89 g g^−1^ (RH30–60–90%)	27 °C and RH80%: 0.6 g g^−1^ (2 min)	[54]
	SMAG	0.7–3.4–6.7 g g^−1^ (RH30–60–90%)	RH30–60–90%: 0.55–3.2–5.8 g g^−1^ (6 h)	[23]
	P(VCL‐co‐AA)/PAN	0.05–0.14–0.5 g g^−1^ (RH30–60–90%)	25 °C and RH80%: 0.27 g g^−1^ (2 h)	[53]
	ZnO hydrogel	0.2–1.2–3.7 g g^−1^ (RH60–90–95%)	—	[27]
	PAM‐CNT‐CaCl_2_	0.69–1.08–1.73 g g^−1^ (RH35–60–80%)	RH35–60–80%: 0.69–1.08–1.73 g g^−1^ (12 h)	[18]
	SPHCs	0.15–0.28–0.9 g g^−1^ (RH30–60–90%)	—	[55]
	PDMAPS‐LiCl	0.62–0.9–1.85 g g^−1^ (RH30–60–90%)	RH30%: 0.62 g g^−1^ (2 h)	[24]
	POG	0.5–2.6–6 g g^−1^ (RH30–60–90%)	—	[62]
	SHPF	0.64–0.96–1.53 g g^−1^ (RH15–30–60%)	RH15–30–60%: 0.64–0.96–1.53 g g^−1^ (2 h)	[25]
Aerogel	PC‐MOF	0.5–1.3–3 g g^−1^ (RH30–60–90%)	—	[98]
	PGF	0.64–3.3 g g^−1^ (RH60–95%)	—	[56]
	G‐PDDA	0.13–0.37–1.01 g g^−1^ (RH30–60–90%)	RH60%: 0.37 g g^−1^ (10 min)	[26]
	LiCl@rGO‐SA	1.5‐3.3 g g^−1^ (RH30–60%)	RH30‐60%: 1.5–3.3 g g^−1^ (3 h)	[21]
	NBHA	0.8 g g^−1^ (RH30%)	RH30%: 0.3 g g^−1^ (3 h)	[22]
	CNF monolith	0.4‐0.8 g g^−1^ (RH30–60%)	‐	[102]
	SMCA	0.5–1–2.5 g g^−1^ (RH30–60–90%)	RH30‐60‐90%: 0.5–1–2.5 g g^−1^ (2 h)	[58]
Foam or sponge	rGO/PI nanosheets	0.25–0.8–2.7 g g^−1^ (RH30–60–90%)	RH30‐50‐70%: 0.25–0.6–1.0 g g^−1^ (4 h)	[71]
	ILCA	0.5–1.15–1.5 g g^−1^ (RH30–60–90%)	RH20‐50‐80%: 0.5–0.9–1.8 g g^−1^ (4 h)	[59]
	IMFCA	0.62–1.4–2.5 g g^−1^ (RH30–60–90%)	RH20‐50‐80%: 0.6–1.0–2.0 g g^−1^ (4 h)	[60]
	NPS	0.035–0.16–0.18 g g^−1^ (RH30–60–90%)	RH40%: 0.21 g g^−1^ (10 min)	[20]
	CNTs‐CILs@cotton rod	0.75–1.2 g g^−1^ (RH 60–90%)	—	[70]

#### 3.2.2 Kinetic Performance

Kinetic sorption performance expresses the change of water uptake with time, which emphasizes the sorbent's practical sorption capacity during a specific process. In SAWH application, sorbent's sorption kinetics determines the practical water uptake within a given time to a certain extent as well as the number of cycles per day. It is a vital indicator for affecting SAWH's systemic water productivity. However, it should be noted that systemic sorption kinetics is not completely dominated by sorbent sorption kinetics but is also affected by systemic condensation efficiency, water recovery efficiency, and the coupling performance between components.^[^ ^63^ ^]^ Different from the static sorption performance, sorbent's sorption kinetics not only rely on the material's intrinsic properties but also have tight relationships with the sorbent's size, shape, and specific operational condition (temperature, relative humidity, and airflow rate).^[^ ^25^, ^64^ ^]^

**Figure 4:**
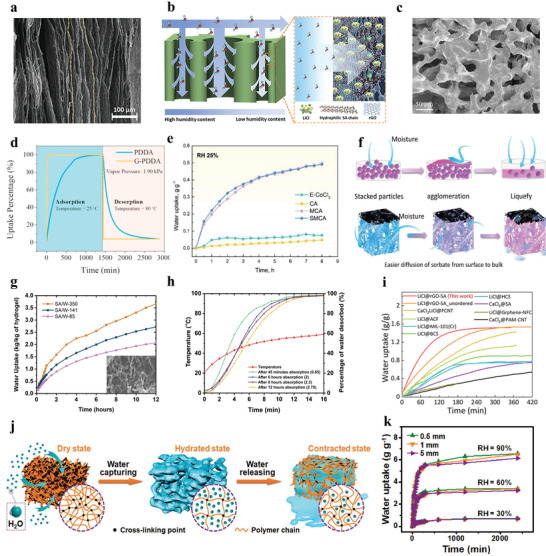
Sorption kinetics of HPPs. a) SEM image of LiCl@rGO‐SA; b) water vapor's transfer process from the ambient to the internals of LiCl@rGO‐SA. c) SEM image of G‐PDDA aerogel; d) water sorption kinetics of G‐PDDA at 25 °C and RH 60%. c,d) Reproduced with permission.^[^ ^26^ ^]^ Copyright 2020, Elsevier. e) Water sorption kinetics of E‐CoCl_2_, MCA, SMCA, and CA at 25 °C and RH 25%; f) comparison of the water sorption process between CoCl_2_ particles and SMCA monolith. e) Reproduced with permission.^[^ ^58^ ^]^ Copyright 2021, Royal Society of Chemistry. f) Reproduced with permission.^[^ ^27^ ^]^ Copyright 2019, Wiley. g) Water uptake of ZnO*_x_* hydrogel with the change of the ratio of surface area to mass, along with the SEM image; h) mass change and surficial temperature of ZnO*_x_* hydrogel during desorption process driven by the halogen lamp (AM 1.5 condition). i) Comparison of dynamic water sorption performance for recently reported novel sorbents that present LiCl@rGO‐SA’ s desirable sorption kinetics. a,b,i) Reproduced with permission.^[^ ^21^ ^]^ Copyright 2021, Royal Society of Chemistry. j) Water sorption and desorption behaviors of SMAG; k) water uptake of SMAGs with different thicknesses during the sorption process at certain relative humidity. j,k) Reproduced with permission.^[^ ^23^ ^]^ Copyright 2019, Wiley.

Considering the material properties of HPP, its sorption kinetics property is directly determined by its heat and mass transfer properties. It denotes that the smaller the resistances of diffusion and permeation are, the better the kinetic sorption performance is. The object with a natural porous structure and large specific surface area causes relatively low transfer resistance for the movement of water vapor molecules.^[^ ^65^ ^]^ Recently, two classical porous structures for HPPs have been reported with desirable heat and mass transfer properties. One is HPP with a vertically aligned nanoscale porous structure, which enables water vapor to predominantly move along the vertical direction and realize a fast sorption rate caused by reducing the tortuosity of water transfer channels (**Figure** **4**a,b).^[^ ^21^ ^]^ The other one is HPP with an interconnected porous structure synthesized via unique gas‐assisted expansion and perforation processes, which is carefully tailored for both effective vapor diffusion and phase transition in the light of the fluid's motion path.^[^ ^65^, ^66^ ^]^ In this case, the interlinked pores benefit the convective flow to easily penetrate the whole structure of HPP, further accelerating vapor's diffusion and moisture's capture.

A scanning electron microscopy (SEM) image of a recently reported G‐PDDA aerogel reveals its highly interconnected macroporous structure (Figure 4c).^[^ ^26^ ^]^ Unlike microporous crystalline sorbents, G‐PDDA aerogel captures water vapor into the bulk, mainly based on the water absorption induced by surface hydrophilic functional groups and capillary condensation within nanoscale pores. Its rate‐determining step for sorption kinetics is the moisture diffusion process from HPPs’ surface to its bulk. Furthermore, an outstanding interconnected structure of G‐PDDA enables numerous water‐captured sites exposed to moisture and facilitates water molecules to transport into HPPs’ internals (water‐captured site denotes a site that can capture moisture and interact with harvested water molecules within the HPP). On the other hand, the thin PPDA film coated on the surface of the rGO skeleton could shorten moistures’ diffusion distance from the ambient to the bulk of G‐PDDA. A corresponding kinetic sorption experiment has demonstrated almost accomplished one sorption–desorption cycle within 20 min by G‐PDDA aerogel (Figure 4d).

Compared with powdery MOFs or granular composites, HPPs’ superior sorption kinetics can be universally ascribed to their porous skeletons. Taking SMCA as an example,^[^ ^58^ ^]^ it reaches water sorption equilibrium drastically faster than the related pure hygroscopic powders E‐CoCl_2_ (Figure 4e). The pure E‐CoCl_2_ particles are usually stacked and present a very limited exposed surface to ambient air. In the initial sorption process, surficial E‐CoCl_2_ particles capture water vapor and form salt crystals, which easily agglomerate and clog subsequent water transport channels. In this case, the internal E‐CoCl_2_ sorbents cannot contact free water molecules in time, further impairing the integral sorption kinetics. On the contrary, uniformly embedding E‐CoCl_2_ into a highly porous skeleton could avoid sorbent's sluggish sorption kinetics (Figure 4f). On the one hand, the interconnected cellulose network can disperse E‐CoCl_2_ particles and amplify the contact probability between hygroscopic factors and water molecules, which encourages the simultaneous work for almost all E‐CoCl_2_ particles. At the same time, the macroporous skeleton shortens the diffusion length of water vapor and the permeation distance of captured liquid water, assuring the constant sorption process without the loss of gas channels.

**Figure 5:**
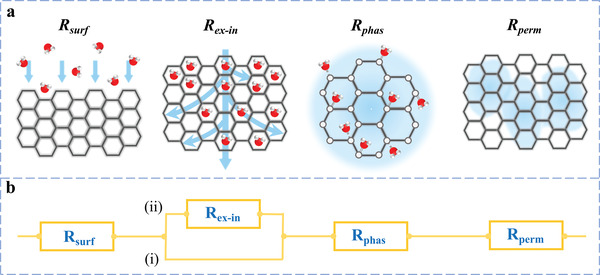
Resistance for water vapor transferring from the ambient to the internal of HPP. a) The schematic diagram for four kinds of mass transfer resistances; b) two kinds of parallel mass transfer paths.

In terms of HPPs’ size and shape, large specific surface area and small thickness are conducive to rapid sorption–desorption kinetics. The ZnO*_x_* hydrogel is a non‐stoichiometric oxide, possessing numerous nanoscale pores and fringed contours.^[^ ^27^ ^]^ The existing enormous surface area and porous structure guarantees the fast water sorption behavior together, while both the surface area and water content have a huge influence on the sorption and desorption rates (Figure 4g,h). Speaking of the water sorption process of HPPs, the captured water molecules generally have two parallel paths (**Figure** **5**a,b): i) water vapor moves from ambient to material's surface, then comes in contact with surficial water‐captured sites and condenses into harvested water, and finally, the liquid water permeates into HPPs’ deeper internal structure. ii) water vapor moves to HPPs’ surface, continuously diffuses into HPPs’ nanoscale pores; then, associates with the wall of internal pores and is formed into liquid water, with the last step being the permeation of liquid water.

Thus, HPPs’ sorption kinetics is mainly controlled by four parts of resistances: the convective transfer resistance from ambient to the external surface of HPP (*R*_surf_), the diffusion transfer resistance from the external surface to the internal surface (R_ex‐in_), the phase transition resistance in the liquefaction process (*R*_phas_), and the liquid transport resistance associated with the internal permeation of captured water (*R*_perm_). In general, the *R*_surf_ mainly depends on external experimental conditions such as ambient temperature, relative humidity, and natural/forced airflow velocity. The *R*_phas_ could reveal the water affinity between HPP and water molecules, which is a small value in the whole resistance system due to the high speed of phase change. Both the *R*_ex‐in_ and the *R*_perm_ are dominated by the size, shape, and structure of HPP, which are larger values that significantly affect the sorption rate. By adjusting HPPs’ specific surface area as large as possible, HPPs’ surface gets closer to its internal, so the transfer paths for both vapor's diffusion and liquid's permeation are shorter. It results in decreased *R*_ex‐in_ and *R*_perm_. In addition, more water molecules tend to migrate following the abovementioned path (i), further alleviating the integral *R*_ex‐in_. However, it must be noted that the size and shape of HPP should be elaborately selected with comprehensive consideration involving the dosage of HPP, the total water productivity, and the utilization efficiency in terms of time and space.

In practical SAWH applications, the environmental conditions are inconsistent. The aim of an SAWH system is to achieve water productivity as high as possible under given experimental conditions. Essentially, the water sorption process is driven by the water vapor partial pressure difference between the surrounding and HPPs’ internals, which correlates with the sorption kinetics. Meanwhile, employing a forced air supply is beneficial to enhance the sorption rate based on the reduction of *R*_phas_ and *R*_surf_ as shown below. On one hand, the sorption process is an exothermic reaction, so timely dissipation of the sorption heat could facilitate subsequent sorption reactions. On the other hand, the high airflow velocity could supplement ample moisture as soon as possible, expediting the migration rate of water vapor from the ambient to HPPs’ external surface. Based on the significance of sorption kinetics, many researchers have reported progress in developing promising sorbents and demonstrated their desirable sorption kinetics. Nevertheless, it is meaningless to describe the sorption rate alone under a certain condition, while a direct comparison of the sorption rate with other sorbents under inconsistent and thus non‐comparable condition is wrong and futile. At present, there is a ubiquitous challenge lacking an accurate and scientific approach to evaluating various sorbent sorption kinetics quantitatively. It is urgent and critical to break down the bottleneck for promoting next‐generation development in aspects of sorption kinetics.

Therefore, here we construct a rational standardized evaluation strategy, to provide a guideline for research related to sorption kinetics. i) As the sorption kinetics is affected by multiple factors such as the material's intrinsic properties, size, shape, and environmental conditions, describing HPPs’ sorption kinetics quantitatively should fix and state all influential variables (Figure 4i). Similarly, comparing sorption kinetics for several sorbents should assure that their size, shape, and experimental condition are all the same. ii) As for micro‐/milligram‐scale HPP samples, a commercial thermogravimetric analyzer (TGA) can be utilized to measure and compare sorbent sorption kinetics. In this case, the size and volume of various HPPs should be completely consistent. A completely dried sample should be placed into a TGA device to conduct the measurement of sorption–desorption kinetics. Note that every time the temperature and humidity procedure as well as the airflow rate should be set the same. iii) As for gram‐scale HPP monoliths, a low‐cost and simple SAWH device can be established using the constant humidity and temperature chamber. The temperature, relative humidity, and air convection should be consistent every time. Moreover, various samples of the same size and volume are placed in the chamber separately, simultaneously recording their mass change over time. When it comes to the desorption rate, a temperature‐controlled electric heater can be adhered with a sample supporter. The same desorption temperature should be set for all samples, while the desorption kinetics for different samples are measured. The gram‐scale measurement for the evaluation of sorption kinetics could be employed to evaluate and predict HPPs’ actual performance in SAWH devices. iv) Based on the evaluation strategy, a relevant database referring to the sorption kinetics properties of various sorbents could be established, which is conducive to a rational selection of HPP for various SAWH applications. Conclusively, such a rational and standardized strategy is meant to guide the development of next‐generation HPPs. In addition, mentioned guidelines could serve as a foundation for international standardization that could be employed for sorption kinetics measurement for all the sorbents.

Focusing on advancing the sorption kinetics, future progress for HPPs could be directed toward optimizing the aforementioned influential factors to achieve promising heat and mass transfer properties. A great example shown in Figure 4j is the SMAG hydrogel which is fabricated by penetrating the hygroscopic polypyrrole chloride (PPy–Cl) into the hydrophilicity‐switchable polymeric network of poly‐*N*‐isopropylacrylamide (PNIPAM).^[^ ^23^ ^]^ The PPy–Cl nanoparticles through their solvation effect absorb and liquefy water vapor rapidly. The absorbed water is then transferred into the internal of PNIPAM's network, in which case, the water‐captured sites on SMAG's surface are available again for follow‐up water absorption. PPy–Cl's unique water sorption mechanism weakens its reliance on the environmental humidity ratio, leading to a small resistance from the ambient to SMAG's external surface (*R*_surf_). Ascribed to the perfect combination of PNIPAM and PPy–Cl, the ultrafast transport and permeation of absorbed water decouple the influence of the *R*_ex‐in_ and the *R*_perm_ on the water sorption kinetics. At the moment, the swelling behavior of SMAG is slower than the water transport, becoming the rate‐determining step for its sorption kinetics. As shown in Figure 4k, the sorption rate of SMAG is insensitive to the change in HPPs’ thickness. The ingenious design of HPP material thus provides an original idea and direction for subsequent optimization.

### 3.3 Desorption Performance of HPPs

As for all sorbents, the desorption process is generally triggered by heating the bulk to urge the water release. Heating the HPP causes a steep increase of local water vapor partial pressure, which is no longer equal to environmental water vapor partial pressure. Hence, the previous equilibrium is broken and the water vapor migrates continuously along water vapor partial pressure difference. From the macroscopic view, the captured water is desorbed and evaporated into its surrounding ambient air. With the accumulation of desorbed water vapor, its dew point temperature tends to be above the condenser wall's temperature; thus, causing vapor condensation on the surrounding wall of the SAWH device. Looking from the perspective of the sustainability, easy availability, and environmental friendliness of solar energy, it is widely utilized to drive SAWH's desorption process. In this review, we mainly introduce and analyze the HPPs which are driven by solar energy. The important indicators that define desorption performance mainly include the desorption kinetics, the required desorption temperature, and the energy consumption for desorption. The desorption kinetics is not only affected by the influential factors illustrated in the previous section but also by HPPs’ thermal conductivity, desorption enthalpy, and the initial water content. In this section, we focus on the attainable desorption temperature and the achievable desorption efficiency.

#### 3.3.1 Influence of Photothermal Property

**Figure 6:**
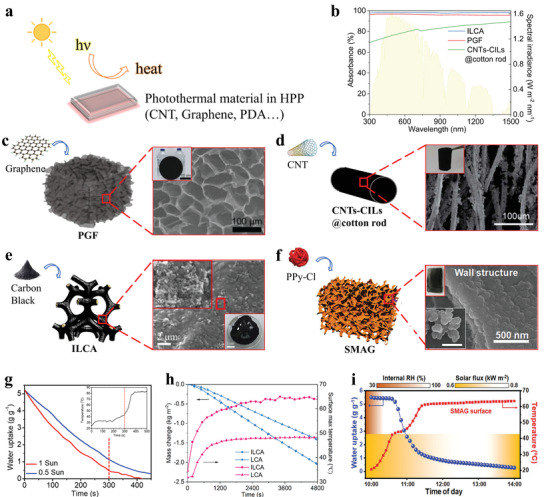
Photothermal materials commonly utilized in HPPs. a) Solar conversion mechanism of photothermal material. b) Sunlight absorption for three kinds of HPPs (ILCA,^[^ ^59^ ^]^ PGF,^[^ ^56^ ^]^ and CNTs‐CILs@cotton rod^[^ ^70^ ^]^). c) The utilization of graphene for the porous PGF fabrication with excellent photothermal properties. d) Impregnation of CNT, forming the intervals of fibers in CNTs‐CILs@cotton rod. Reproduced with permission.^[^ ^70^ ^]^ Copyright 2021, Royal Society of Chemistry. e) The utilization of carbon ink containing carbon nanospheres on the surface of ILCA's fibers. f) The embedment of hygroscopic PPy–Cl on the nano surface; thus, obtaining the black SMAG. g) Water uptake and surficial temperature of PGF during the desorption process under 0.5 sun or 1 sun. c,g) Reproduced with permission.^[^ ^56^ ^]^ Copyright 2020, Wiley. h) Mass change and surface maximum temperature of LCA and ILCA under a one‐sun desorption process. e,h) Reproduced with permission.^[^ ^59^ ^]^ Copyright 2021, Elsevier. i) Water uptake and surficial temperature of SMAG under the natural sun. f,i) Reproduced with permission.^[^ ^23^ ^]^ Copyright 2018, Wiley.

In the case of the photothermal‐based HPPs, the desorption temperature is related to both the solar absorber's temperature and the thermal resistance from the solar absorber to the sorbent material. Similar to previously reported powdery MOFs and granular composites, a single solar absorber should be adhered to sorbents for achieving photothermal properties.^[^ ^67^ ^]^ This configuration introduces a thermal contact of a layer, which in turn increases systemic complexity. Profiting by HPPs’ form of a monolith, it not only presents excellent compatibility and easy composability in the SAWH system but also enables in situ heating by combining photothermal materials with the sorbent materials during the fabrication process. Carbon‐based solar absorbers have advantages in terms of cost, processibility, and photothermal conversion, and have become the prevailing photothermal materials for SAWH application. Their basic mechanism for generating heat is derived from the thermal vibration of molecules (**Figure** **6**a). There are numerous delocalized *π* bonds formed from unbound free electrons which exist in six‐membered rings‐based lattices of carbon‐based materials. After absorbing incident photons with matched energy, the *π* electrons are excited to a higher molecular orbital. The electrons with an excited state are highly unstable, so the relaxation process easily occurs and leads to the electron's ground state, which induces the energy release in the form of heat.^[^ ^68^, ^69^ ^]^ Due to the strong and broadband light absorption, carbon‐based materials have been developed as outstanding candidates for exerting the photothermal property. Commonly‐used carbon‐based absorbers mainly involve graphenes, carbon nanotubes, and several amorphous carbons such as graphite and carbon black. Attributed to their dominant potential in photothermal applications (Figure 6b), they have been widely utilized to localize heat in bulk HPP.^[^ ^56^, ^59^, ^61^, ^70^ ^]^

Both normal and reduced graphene present a 2D flake structure with a hydrophilic surface and enormous surface area. Employing some fabrication methods such as 3D printing, crosslinking, and freeze‐drying, they are widely assembled into a porous structure of HPP to induce the solar‐driven desorption process.^[^ ^71^ ^]^ The typical HPP is the microporous PGF aerogel,^[^ ^56^ ^]^ which is easily fabricated based on the porous sodium polyacrylate and graphene framework through a convenient freeze‐drying method (Figure 6c). Numerous surficial oxygen functional groups are responsible to capture water vapor through hydrogen bonding. The extraordinary photothermal property of PGF derived from black graphene endows itself with considerable desorption performance: under one sun, the desorption temperature can reach ≈80 °C within 7 min. Assisted by the effective gas transport channel and enlarged solid–gas contact area, the desorption process can be accomplished within 7 min under environmental conditions (Figure 6g). A carbon nanotube (CNT) is composed of several concentric circular tubes with the composition of a hexagonal arrangement of carbon atoms. It belongs to 1D quantum material with superior photothermal performance. Adding a small amount of suspension containing CNT to HPPs’ reaction solution is a frequently‐used method to attach HPP with excellent photothermal properties, such as the CNTs‐CILs@cotton rod (Figure 6d).^[^ ^70^ ^]^ In addition, cheap carbon black (CB) nanoparticles can be uniformly dispersed into HPPs’ porous skeleton, providing admirable photothermal properties. Chinese black ink is a low‐cost, easily available, and environmentally friendly carbon‐contained material, which is also a promising candidate through a facile spray coating or dipping method.^[^ ^72^, ^73^ ^]^ ILCA is a typical HPP, fabricated by impregnating carbon nanospheres into the hierarchical porous skeleton of the natural loofah sponge (Figure 6e).^[^ ^59^ ^]^ Meanwhile, both the binders in a carbon ink and the ion crosslinking of calcium alginate are conducive to the stable existence of carbon nanospheres on loofah's stems. During the initial 20 min, the desorption temperature can reach ≈60 °C under one sun. Meanwhile, the desorption of ILCA is 1.4 times faster than that of the sample without carbon nanospheres (Figure 6h). Except for the abovementioned carbon‐based photothermal materials, some polymers such as PPy–Cl and polydopamine (PDA) are widely mixed in the porous network of HPPs as well.^[^ ^74^, ^75^ ^]^ They obey the same working principle to play a role in absorbing sunlight and motivating the water release. The promising thermal‐responsive SAMG embeds PPy–Cl in PNIPAM's skeleton, making use of PPy–Cl's high photothermal property to drive SMAG's desorption (Figure 6f). Under the sunlight after 1 h, the surficial temperature of SMAG is up to 63 °C, and the absorbed water is rapidly oozed out (Figure 6i).

#### 3.3.2 Influence of Thermal Conductivity

HPPs’ thermal conductivity has an important influence on HPPs’ heat transfer properties. Considering that the sorption process is an exothermic reaction, the sorption heat can be timely released to the ambient due to the relatively slower sorption rate during the long‐time sorption process. Generally, HPPs’ sorption kinetics is loosely affected by its heat transfer properties. In contrast, the desorption process driven by thermal energy has a tighter relationship with the heat transfer process. During the desorption process, HPP absorbs sunlight and transforms it into heat. In addition, the desorption process can be driven also by any other form of heat. Internal heat conduction transfers heat from HPPs’ top surface to its internal; thus’ maintaining HPP at a uniform desorption temperature. In the meantime, heat convection takes place with the surrounding air. Both convection and heat radiation enable a thermal pathway between the condenser and sorbent at elevated temperatures. The parasitic heat losses, as a result, are undesirable side effects of HPPs’ desorption. Meanwhile, HPPs’ thermal conductivity determines its internal heat conduction process directly, and it affects the heat convection between HPP and surrounding air to a certain extent. Thus, thermal conductivity is very pivotal for sorbent's desorption process. High thermal conductivity enables a quick rise of HPPs’ desorption temperature uniformly within the bulk of a sorbent. On the other hand, the heat transfer between HPP and the surrounding air is predominantly not influenced by the thermal conductivity of an HPP, whereas the convective heat transfer coefficient has a significantly larger effect. However, in the sealed desorption chamber with small air gaps, vapor diffusion is dominant, while air convection is weak. Higher thermal conductivity of an HPP is highly beneficial to the desorption process, while the parasitic heat losses are not influenced by this quantity.

Previous studies employed granular sorbents that were usually stacked, leading to increase of the material's dosage in practical SAWH applications. In this case, the intergranular space is filled by nearly stationary air, which increases the overall thermal resistance of a material.^[^ ^63^, ^76^ ^]^ During the desorption process, the captured water is desorbed into water vapor and ejected into the ambient. The existence of air thermal resistance degrades sorbent's thermal conductivity, further disturbing uniform desorption temperature. In comparison, HPP is composed of continuous solids and liquids, where the overall heat transfer resistance along the interconnected cross‐linked network is lower due to the lack of air inside the network. This fact assures HPPs’ superior thermal transfer properties.

In light of enhancing sorbent's thermal conductivity, the dispersion of a material with high thermal conductivity is a commonly utilized approach. Carbon‐based materials including graphite, carbon fiber, carbon foam, and expanded graphite are promising additions for enhancing sorbent's thermal conductivity.^[^ ^68^ ^]^ Granular sorbents, carbon nanotubes, and graphene oxide flacks are commonly used to enhance thermal conductivity. Unfortunately, the air gap unavoidably exists between pristine sorbent and the added carbon‐based material, which further leads to diminished improvement of HPPs’ thermal conductivity. At the same time, the addition may block vapor's diffusion channels inside the granular sorbent; thus, worsening the sorbent's mass transfer properties. These problems are naturally absent in HPPs benefiting from their excellent network structures, in which carbon‐based materials can be in situ impregnated or directly synthesized into the polymer chains. It has been reported that adding a small percentage of carbon‐based material not only cannot sacrifice HPPs’ sorption kinetics but also exhibits an additional improvement in sorption rate and water uptake.^[^ ^59^, ^61^ ^]^ This also proves the huge advantages of HPP compared to the granular sorbents.

#### 3.3.3 Desorption Efficiency

The typical operational mode of a solar‐driven SAWH device consists of two steps.The sorption process occurs at night, while the desorption process takes place in the daytime. In comparison to the sorption rate, the desorption rate is higher, which is ascribed to the relatively larger water vapor partial pressure difference between HPPs’ internal and surroundings during the desorption process. It triggers the familiar phenomenon, in which the desorption process can be completed in less than 8 h, which causes a surplus of solar resources in the daytime. To fully employ the available solar energy in order to gain optimal SAWH performance, matching the time between the sorption and desorption process is pivotal.^[^ ^64^, ^77^ ^]^ For a passive SAWH device, utilizing a batch or continuous desorption process could be utilized to improve solar utilization efficiency during the entire daytime.^[^ ^21^ ^]^ Several HPP‐based sorbent beds can sorb moisture from the atmosphere fully at night. During the daytime, each bed could generate freshwater by the so‐called batch‐alternating process in the daytime. In this case, 6–8 batch processes could be performed per day, with high time and energy utilization as well as large water yield.^[^ ^78^ ^]^

## 4 Optimization of an HPP‐Based SAWH System

Indicators used for evaluating the solar‐driven HPP‐based SAWH system's performance mainly include i) gravimetric water uptake, ii) volumetric water uptake, iii) total water productivity, and iv) water uptake per projected sunlight absorber area. These indicators depend on many factors, such as the sorption–desorption performance of the sorbent bed, the condensation performance dominated by the condenser, and the collection efficiency determined by the condenser and water collector. The rational design of every component is the direction toward enhanced systemic water harvesting performance. In this section, we pay close attention to the optimization of HPP‐based sorbent beds aiming at achieving high water sorption capacity and thorough desorption capacity. Meanwhile, we provide insights to fill the gap in recognizing the sources and potential solutions that highly affect SAWH performance: optimization of the coupled design that includes all major components to realize a high ratio of condensed water to desorbed water and larger solar conversion efficiency.

### 4.1 Optimization of an HPP‐Based Sorbent Bed

**Figure 7:**
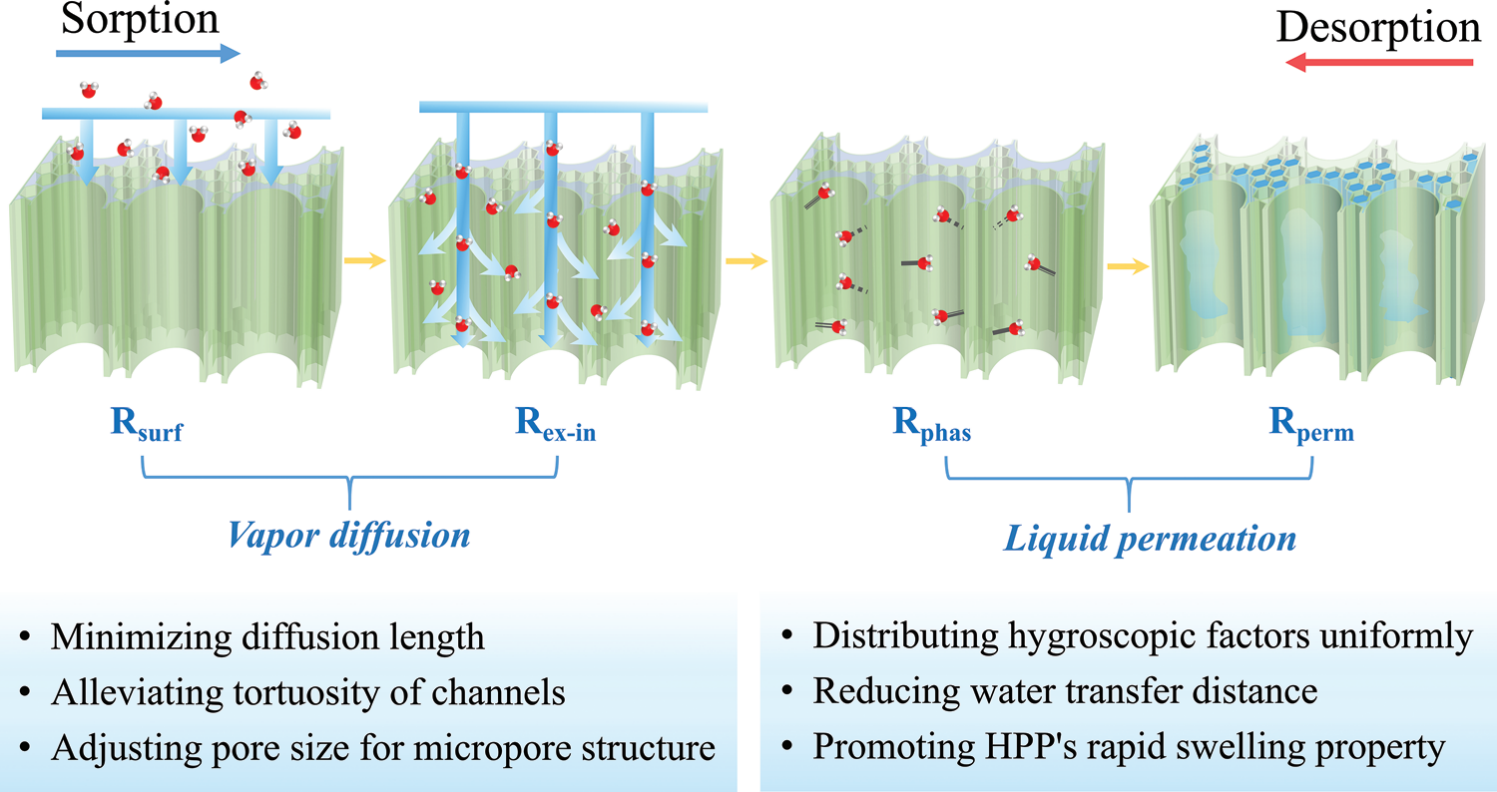
Schematic illustration of mass transfer in HPP‐based sorbent bed. The four steps indicate the mass transfer resistances successively during sorption and desorption processes. First, water vapor moves from ambient air to the external side of HPP through natural/forced convection (*R*_surf_). Second, water vapor diffuses from the external to the internal of HPP (*R*_ex‐in_). Third, water vapor molecules react with the active water‐captured sites of HPP to form absorbed/adsorbed water (*R*_phas_). Finally, the captured water permeates into the deeper location of HPP through its internal channels (*R*_perm_).

An HPP in the form of a monolith can be flexibly assembled in a SAWH device and regarded as the sorbent bed. As such, the optimization of the sorbent bed is complementary to the structural design of an HPP. According to previous content in Section 3.3, the relevant optimization strategy for enhancing the heat transfer properties of sorbent bed has been discussed: dispersing carbon‐based material in HPPs’ network to obtain both high thermal conductivity and excellent photothermal properties simultaneously. Herein, the structural design of an HPP to realize desirable mass transfer property is presented. Corresponding to the description in Section 3.2, there are four layers of mass transfer resistances existing during the sorption and desorption process shown in **Figure** **7**: *R*_surf_, *R*_ex‐in_, *R*_phas_, and *R*_perm_. Except for the external experimental conditions, the *R*_ex‐in_ and the *R*_perm_, both of which are dominated by the structural features of the HPP‐based sorbent bed, are emphatically analyzed and illustrated here.

#### 4.1.1 Vapor Diffusion From Surface to Internals of an HPP

The sorption process is driven by the water vapor partial pressure difference between the ambient and the internals of an HPP. Therefore, numerous water vapor molecules migrate from ambient to the HPP following two kinds of parallel paths shown in Section 3.2.2: i) first, the molecules associate with surficial hygroscopic factors of HPP and subsequently form liquid water,^[^ ^47^ ^]^ which penetrates inside HPPs’ network.^[^ ^79^ ^]^ ii) Subsequent water molecule continues the diffusion process until it bonds with unoccupied water‐captured sites. In general, HPPs have a uniform microscale porous structure, based on which there are lots of nanoscale pores or folds.^[^ ^27^ ^]^ Water vapor molecules with a diameter of ≈0.4 nm can transfer through these microscale or nanoscale channels successfully. Then they come in contact with the wall of internal interconnected pores and bond with hydrophilic functional groups. The captured water adheres to HPPs’ inner wall as a layer of water film, which also tends to penetrate inside. As the water sorption process continues, supplementary water molecules diffuse in the water film‐attached channel. During diffusion, they suffer from the viscous friction with the water film on the wall and the collision between moving water molecules.^[^ ^80^, ^81^ ^]^ As the average free path of a water molecule is ≈85 nm, the majority of water molecules can avoid colliding with the wall of HPPs’ microscale channels.^[^ ^82^ ^]^ Water vapor predominantly flows through in the form of a gaseous flow, and the resistance is dominated by the collisions between water molecules rather than viscous friction. As for HPPs’ nanoscale pores with a diameter below 85 nm, the resistance is focused on viscous friction, and the transfer of water vapor is referred to as Knudsen flow.^[^ ^63^ ^]^ Generally speaking, the collision between moving water molecules is a natural phenomenon, which is inevitable and cannot be slowed down. Thus, the reduction of the proportion of water molecules that experience long‐distance diffusion is feasible, leading to reduced mass transfer resistance. In other words, HPPs’ structure allows water molecules to find unoccupied water‐captured sites as soon as possible and liquefy them to come into the next permeation process.

The specific measures oriented at enhancing vapor diffusion from surface to internals of an HPP are: i) Splitting and thinning the HPP material by constructing macro millimeter‐scale porous structures to minimize the diffusion length of water molecules.^[^ ^83^ ^]^ Colloquially, the water molecules captured on HPPs’ external surface have the shortest migration distance. Using such a measure, more water vapor molecules are in direct contact with bare active water‐captured sites directly instead of molecules moving in narrow and sinuous channels. ii) As for the inside water‐captured sites without direct exposure to ambient moisture, rationally constructing the porous structure can be adopted to alleviate the tortuosity or conform with the water molecule's natural migration.^[^ ^65^, ^66^ ^]^ This method mainly reduces the viscous friction with the walls of pores and lowers the collision between water molecules that are in motion. iii) Constructing a uniform microscale porous structure (the diameter of pores is generally above 0.1 µm) can provide ample channels for free diffusion of water vapors.^[^ ^37^ ^]^ This can lower the proportion of water molecules required to be in contact with water film on the pore walls of HPP. In addition, building nano‐sized rough structures is helpful to enlarge the internal surface area, further facilitating the quick capture of more water molecules in limited space and time scales.

As a result, establishing a hierarchical structure with nanoscale, microscale, and millimeter‐scale pores or constructing a porous form satisfying the water molecule's natural migration principle can yield an HPP with excellent mass transfer properties. In this aspect, Xu et al. built an excellent HPP named LiCl@rGO‐SA with a vertically aligned and cascade porous structure, which exhibits fast sorption–desorption kinetics even in low humidity regions.^[^ ^21^ ^]^ Similarly, Deng et al. prepared a unique HPP named ILCA with a hierarchical porous topological structure and demonstrated its excellent SAWH performance in wide humidity ranges.^[^ ^59^ ^]^ LaPotin et al. developed a dual‐stage SAWH device to improve the energy utilization efficiency, in which the condensation heat from the first stage is used to drive the desorption process in the second stage.^[^ ^84^ ^]^ Potentially, the dual‐stage design used for sorbent bed also increases zeolite's dosage in limited projected area without sacrificing sorbent's sorption kinetics per mass. This idea of designing a multi‐layer sorbent bed could be learned and exploited in the design of HPP. Both the sorption‐tree design proposed by Deng et al.^[^ ^60^ ^]^ and the honeycomb sorbent bed designed by Wang et al.^[^ ^85^ ^]^ are maximizing both the sorption–desorption kinetics and the sorbent material's amount via 3D design in practical SAWH system.

For granular sorbents, relatively large interparticle porosity for the sorbent bed as well as small size for sorbent particle is commonly used to achieve effective mass transfer properties. However, high interparticle porosity leads to low packing density and enormous volume of a sorbent bed, further extending the overall diffusion length that postpones the sorption equilibrium.^[^ ^63^ ^]^ If using a thin layer of a sorbent bed, the limited dosage of sorbent material will restrict the total water productivity for the practical SAWH device.^[^ ^86^ ^]^ This represents a dilemma between the fast sorption–desorption kinetics and high total water productivity in the SAWH system using granular sorbents. This problem can be eliminated in HPP using advanced optimization strategies for the HPP‐based sorbent bed. For SAWH, the ultimate volumetric water uptake is close to 1 g cm^−3^. The number is calculated by assuming that all space of HPP is filled with captured water regardless of the volume occupied by HPPs’ matrix. It is exactly the density of water. Unlike the granular sorbents that sacrifice the volume due to the interparticle void, HPPs with cascade porous structures retain captured water in all pores relying on the inherent water‐retaining property or capillary force.^[^ ^21^ ^]^ Simultaneously, the light porous skeleton of HPP usually assists the captured water to permeate inside HPP, further enhancing the water‐retaining capacity.

#### 4.1.2 Water Permeation Inside the HPPs’ Internal

Driven by the local chemical potential difference between wet and dry zones within HPP,^[^ ^79^, ^87^ ^]^ the captured water is redistributed in HPPs’ porous network through the permeation process. By considering various substrates for HPPs, there are two kinds of permeation processes. In hydrogel‐based HPP, the liquid water is permeated in the nanopores, which leads to hydrogel's swelling and deforming behaviors. Inside the aerogel‐based or foam‐based HPPs, harvested water is maintained and stored in HPPs’ porous structure owing to the microspores’ capillary forces. As there is no expansion and a consequent deformation, the maximum water capacity is determined by the volume of the HPPs’ skeleton.

HPPs’ permeation behavior is mainly affected by the porosity, liquid permeation direction, and the form of a porous structure, which is similar to the gas diffusion process. The transfer resistance is composed of the viscous force between liquid water molecules, the friction forces between liquid water and the network's molecular chain, and the collisions with solid essences within HPP. Analogously, shortening the penetration distance of liquid water as much as possible also benefits to accelerate the mass transfer process. According to the different existing form of HPPs’ hygroscopic factors, the corresponding optimization strategies for liquid water transfer need to be discussed separately. For HPP which includes dissociative hygroscopic factors, both the free hygroscopic factors and the captured water are filled in HPPs’ porous structure during sorption. Preventing the inhomogeneous distribution of hygroscopic factors from clogging the transfer channels for both water vapor and captured liquid water is the key point for optimization. For HPP on whose polymer chains the hygroscopic factors are implanted, the active water‐captured sites work by liquefying moisture and transferring it into the porous network.^[^ ^88^ ^]^ In this case, assuring the sorption rate of surficial hygroscopic factors on HPP is the critical point. It is an effective approach to reduce the percentage of water vapor required to conduct long‐length transfer, further realizing fast sorption kinetics.^[^ ^62^ ^]^ Meanwhile, accelerating the permeation process of captured water by promoting HPPs’ swelling capacity could bring a large improvement for liquid permeation.^[^ ^23^ ^]^

### 4.2 Coupling Design of All Components

The optimizations of the condenser, the solar absorber, and the coupling design among all components are equivalently important to obtain high SAWH performance in comparison to the advancements on the material–HPP level. In this section, we will propose novel coupling designs that span among all components.

#### 4.2.1 Coupling Between Solar Absorber and Sorbent Bed

**Figure 8:**
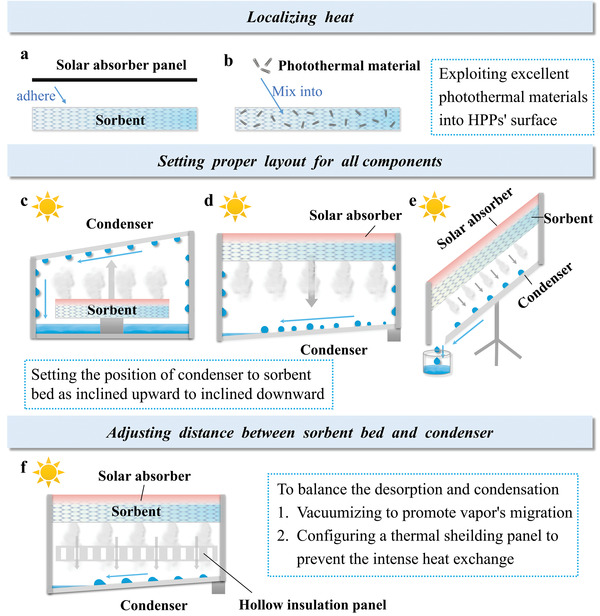
Schematic illustration showing possible structural optimizations for HPP‐based SAWH system. a) Combination of HPP‐based sorbent bed and the solar absorber panel; b) HPP containing the photothermal material. c) “Up–Down” layout of “Condenser‐Sorbent bed”; d) “down–up” layout of “Condenser‐Sorbent bed”; e) optimized inclined structure for next‐generation HPP‐based SAWH system to balance the solar absorptance and the condensation performance. f) Optimized strategy for enhanced vapor transport by adding a hollow insulation panel to balance systemic heat and mass transfer properties.

For the HPP‐based SAWH device driven by solar energy, solar absorber is combined with the sorbent bed which includes two categories: one is that the sorbent bed is adhered to a solar absorber panel; the other one is the direct mixture of photothermal material into HPP to realize the in situ heating (**Figure** **8**a,b). For the first one, the heat transfer between the two components should be carefully taken into account.^[^ ^89^ ^]^ Usually, employing an air interlayer above the solar absorber inhibits its heat losses toward other objects. Moreover, the heat conduction between the solar absorber and the sorbent bed should be enhanced. For instance, an increased thermal conductivity of the sorbent bed could be realized by spraying thermally conductive silicone grease to eradicate the air thermal resistance layer between the solar absorber and the sorbent bed. In addition, if the required desorption temperature cannot be realized under current solar irradiation intensity, the structural design through setting both a larger solar absorber surface and a smaller desorption area is favorable to achieving concentrated thermal energy for raising HPPs’ temperature.^[^ ^90^ ^]^ For the second one, endowing HPP with high photothermal properties is a promising solution for reducing systemic complexity and downsizing the usage of heat insulations, which is also widely utilized among SAWH systems in the recently reported literature. However, the thermal insulation on the other sides of the sorbent bed is significant in order to reduce parasitic heat losses to the condenser or other objects as much heat as possible on HPP. Furthermore, modifying HPP with both excellent photothermal properties and low thermal capacity is beneficial to the advancement of desorption temperature, further boosting the desorption efficiency.

#### 4.2.2 Location and Feature of Condenser

In a practical SAWH device, the condenser is horizontally arranged in general, which is vertically parallel to the sorbent bed. Commonly, the layout of the condenser toward the sorbent bed is an up–down arrangement or down–up arrangement (Figure 8c,d). When the condenser is above the sorbent bed, it should be transparent to allow sunlight transmission. Under the sunlight, moisture is desorbed and diffuses upward; then, releases heat and condenses into liquid. The condenser water is attached to the wall of condenser, which impairs the sunlight transmittance for the HPP‐based sorbent bed. In addition, the water droplets have a high absorptance in the near‐infrared wavelength range, which accounts for a significant proportion of solar irradiation in the full spectral range. On the one hand, the condensed water harvests a part of available solar energy and decreases the effective solar conversion efficiency. On the other hand, after attaining near‐infrared energy, there is a risk of re‐evaporation, leading to the high relative humidity above the sorbent bed that inhibits desorption. The multiple effects will thus impair both the desorption efficiency and condensation efficiency.

When the desorbed moisture condenses beneath the sorbent bed, the aforementioned problems can be averted. The condensed water has little influence on the photothermal property and solar conversion efficiency of the SAWH system. However, as the density of water vapor is less than the air, desorbed water vapor tends to migrate upward, driven by buoyancy. The desorbed moisture has to overcome its gravity to diffuse downward, in which case the migration resistance from the sorbent bed to the condenser is unavoidably amplified.

There are several existing problems in the above two kinds of layouts. Thus, a superior structural design is proposed here, where the whole system is arranged in an inclined layout instead of the previous vertical layout (Figure 8e). The relative location of the sorbent bed toward the condenser is set as an inclined up–down arrangement. In this way, the influence of condensed water on sunlight absorption can be eliminated, while the migration resistance of desorbed water vapor can be alleviated. It is worthwhile to mention that there are many auxiliary measurements recently proposed aimed to improve both condensation efficiency and collection efficiency. For example, equipping the condenser with fins could enhance the heat transfer performance and expedite the release of condensation heat. Rationally shortening the length of the condenser could avoid a weak collection rate from a large film of a condensate.^[^ ^91^ ^]^ Modifying the surface structure of the condenser with mixed hydrophilic and hydrophobic properties enables water droplets to drop off timely without affecting the coalescence and growth of water droplets.^[^ ^92^ ^]^ Similarly, creating the surface using the condensation mode known as “jumping droplet condensation” is a promising approach to realize the rapid shedding of condensed water; thus, improving the condensation and collection processes.^[^ ^93^ ^]^

#### 4.2.3 Coupling Between Condenser and Sorbent Bed

In the desorption–condensation process, the water vapor with elevated temperature is desorbed from the sorbent bed and diffuses slowly toward the condenser. Finally, the water vapor comes into contact with the cold wall of the condenser, releasing the latent heat of phase change and turning into water droplets. During the whole process, the water vapor evaporation and condensation have a tight relationship with the desorption temperature and condensation temperature, respectively. At the same time, the water vapor migration route can be seen as the bridge linking the sorbent bed and the condenser, which is highly connected with the systemic heat and mass transfer properties. Therefore, it is pivotal to optimize the distance between the two components, aiming at achieving high SAWH performance.

When the distance is too long, the thermal resistance between the sorbent bed and condenser will increase, leading to their relatively large temperature difference. However, longer distance also introduces larger mass transfer resistance—for the water vapor transport between the sorbent bed and the condenser. Within the sealed chamber with a relatively small air gap; thus, without natural air convection, it is a challenging condition for moisture migration as it only depends on the water vapor's partial pressure. In this case, the moisture may stagnate and appear with high relative humidity on the HPPs’ surface, further retraining subsequent desorption behavior. If the distance is too short, the water vapor diffuses rapidly. Nevertheless, due to heat transfer dependency on the distance between the sorbent bed and the condenser, both the heat conduction and heat radiation are enhanced from the hot sorbent bed to the cold condenser. Correspondingly, the temperature difference decreases, which is adverse for the whole desorption–condensation process.^[^ ^91^ ^]^ The water vapor flow resistance is easily neglected in the design. The specific volume of water vapor is relatively large: for 1 g of condensed water, 1 m^3^ of moist air, consisting of dry air and water vapor desorbed at 80 °C has to condense. If the flow duct connecting the desorption bed and condenser has a small diameter or too long distances, the condensation of desorbed water could be challenging.

To cope with the above troubles, setting an appropriate distance between the two components is urgent and crucial for balancing SAHW's heat and mass transfer properties. In addition, here we propose some other strategies: i) A thermal shielding panel with numerous hollowed‐out holes can be equipped between the sorbent bed and the condenser, which requires low thermal conductivity and high radiation reflectivity (Figure 8f). On the one hand, it reduces the heat conduction and radiation from the sorbent bed to the condenser, maintaining a suitable temperature difference between the two components to promote water evaporation and condensation. On the other hand, the water vapor can pass through the holes successfully without augmenting the mass transfer resistance. ii) During the desorption–condensation process especially in the beginning, intermittently pumping air out of the sealed SAWH chamber reduces the percentage of non‐condensable gas to a great extent. Such a measure amplifies the water vapor partial pressure difference; thus, elevating the water vapor migration.

## 5 Conclusion and Outlook

**Figure 9:**
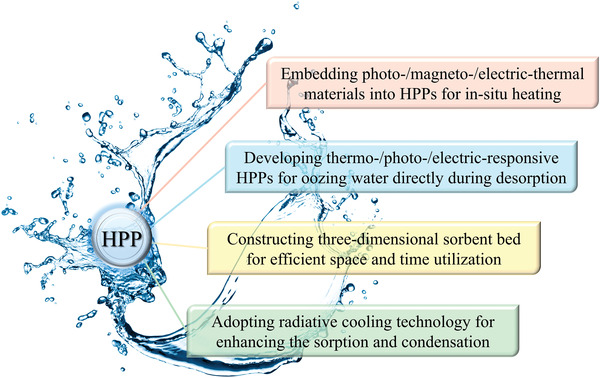
Outlook related to the development of next‐generation HPPs.

In this review, we introduced HPPs’ recent development related to the sorption mechanism, working principle, performance, and application. The corresponding optimization strategies for the HPP‐based sorbent bed and the coupling design of the HPP‐based SAWH system have been elucidated as a guideline for next‐generation SAWH systems. On the way to marketization, several novel outlooks have been brought into the spotlight for the development of next‐generation HPPs from the perspective of energy source, desorption mode, water productivity, and systemic efficiency (**Figure** **9**).

### 5.1 In Situ Heating Method

Almost all existing HPP‐based SAWH systems realize water desorption driven by solar energy, which is infeasible and ineffective in some arid areas without ample sunlight. Therefore, to broaden its application, other methods such as electrical heating should be considered. Similarly, rather than configuring an electrical heating panel for the desorption process, embedding highly electric conductive parts into HPPs is superior to achieving uniform heat localization. Looking at the seawater desalination field, copper wires or electric conductive graphene could be embedded in the internal evaporators to realize the in situ electrical heating function.^[^ ^94^, ^95^ ^]^ This exhibits excellent water production performance even in weak sunlight, achieving all‐day highly efficient water evaporation. Besides, in the case of obtaining considerable water production with large doses of HPP, eddy current heating under an alternating magnetic field is another possible choice to drive the desorption process uniformly via the metal material embed process. Tao et al. demonstrated the inclusion of the metal foam into MOF to trigger sorbent's rapid release behavior of harvested atmospheric water.^[^ ^96^ ^]^

### 5.2 Rapid Response Property

Apart from searching for desirable energy resources to supply the desorption heat, innovating in the adoption of various desorption modes is also insightful for energy‐saving SAWH systems. Recently, several polymers with thermal responsive hydrophilicity–hydrophobicity switching properties such as PNIPAM, hydroxypropyl cellulose (HPC), and poly *N*‐vinylcaprolactam (PVCL) are utilized in the preparation process of HPPs, endowing them with the thermo‐responsive behavior.^[^ ^97^, ^98^, ^99^ ^]^ Generally, during the water sorption process, the HPP captures and liquefies ambient moisture. During the subsequent desorption process, a small elevation of temperature triggers HPPs’ chains to shrink drastically and squeeze out liquid water. Compared with the conventional desorption process, HPPs’ controllable change in water affinity with a small temperature change enables it to ooze liquid water directly, leaving out further condensation of water vapor. The atmospheric moisture thus experiences only one phase change, without requiring a cold source to assist the release of condensation heat. For an HPP‐based SAWH system, fabricating HPP with thermo‐responsive property to assemble it into the device is a potential approach to harvest atmospheric water rapidly and simply, leading to decreased systemic complexity. In addition, electric‐responsive or photo‐responsive HPPs could be synthesized and developed in the future in parallel; thus, making SAWH's application more extensive, prominent, and efficient.^[^ ^100^, ^101^ ^]^

### 5.3 Structural Design of HPP‐Based Sorbent Bed

In line with the optimization of the HPP‐based sorbent bed introduced in Section 4, the structure of the HPP monolith plays an important role in systemic sorption kinetics, further affecting the total water productivity of the SAWH system. Advanced structures of an HPP, such as interconnected porous structure and hierarchical porous structure are highly beneficial for the advanced sorption performance of HPPs. At present, the frequently‐used preparation method for constructing HPP monoliths is the freeze‐drying method after the HPPs’ crosslinking process. Recently, 3D printing has emerged as a prominent method to construct required complicated objects and sensors in other mechanical fields. It demonstrates a huge potential to exploit this technology in HPPs’ fabrication process to obtain ideally porous monoliths. First, a physical model can be constructed via sorption kinetics simulation to understand the ideal porous structure of HPP in certain applied situations. Then, the required ingredients, such as printing ink are selected for the 3D printing process according to the predicted physical model. Its huge advantage is that the elaborate structural design can come true, attributed to the flexibility and scalability of 3D printing, which is crucial for the development of next‐generation HPPs with superior structures.^[^ ^102^, ^103^ ^]^ In terms of the sorbent bed, increasing the sorbent material's dosage is quite significant to obtain substantial systemic water productivity in a limited volume. However, too thick and large sorbents are inadvisable for rapid sorption kinetics and high water uptake per day. Herein, lots of simple 3D structural arrangements can be employed to deal with the problem: adopting multi‐layer material's layout or spatial sorbent bed for HPP is feasible and effective for HPP‐based SAWH system in the limited projected systemic area.

### 5.4 Hybrid System Approach

In terms of systemic performance for SAWH devices, lots of advanced technologies can be supplemented for more effective water harvesting. For example, radiative cooling technology can be exploited to enhance systemic water harvesting performance by assisting the exothermal sorption process and subsequent condensation process.^[^ ^64^ ^]^ For the water sorption process, the radiative cooling materials can be configured with an HPP‐based sorption bed to expedite the dissipation of sorption heat, further advancing the sorption kinetics. For the desorption–condensation process, the release of condensation heat dominates moisture's condensation efficiency, while the heat transfer mainly relies on the natural convection with ambient air to some extent. Using a radiative cooling panel on the wall of the condenser is beneficial to broaden the exothermal channels by emitting long‐wave radiation and reflecting almost 100% of the solar energy. Moreover, radiative cooling technology can be realized passively, for example, without any additional energy consumption, which caters to the sustainable development of SAWH technology.

## Conflict of Interest

The authors declare no conflict of interest.
